# Does Higher Intensity Increase the Rate of Responders to Endurance Training When Total Energy Expenditure Remains Constant? A Randomized Controlled Trial

**DOI:** 10.1186/s40798-023-00579-3

**Published:** 2023-05-20

**Authors:** Marcel Reuter, Friederike Rosenberger, Andreas Barz, Andreas Venhorst, Laura Blanz, Anne Hecksteden, Tim Meyer

**Affiliations:** 1 Insitute of Sports and Preventive Medicine, University of Saarland, Saarbrücken, Germany; 2 German University of Applied Sciences for Prevention and Health Management, Saarbrücken, Germany; 3 Department of Medical Oncology, National Center for Tumor Diseases (NCT), Heidelberg, Germany; 4 Institute of Psychology and Sport Science, Leopold-Franzens-University of Innsbruck, Innsbruck, Austria

**Keywords:** Aerobic fitness, ${\dot{\text{V}}}$O_2max_, HIIT, Energy expenditure, Endurance training, Nonresponder, Response

## Abstract

**Background:**

Standardized training prescriptions often result in large variation in training response with a substantial number of individuals that show little or no response at all. The present study examined whether the response in markers of cardiorespiratory fitness (CRF) to moderate intensity endurance training can be elevated by an increase in training intensity.

**Methods:**

Thirty-one healthy, untrained participants (46 ± 8 years, BMI 25.4 ± 3.3 kg m^−2^ and ${\dot{\text{V}}}$O_2max_ 34 ± 4 mL min^−1^ kg^−1^) trained for 10 weeks with moderate intensity (3 day week^−1^ for 50 min per session at 55% HR_reserve_). Hereafter, the allocation into two groups was performed by stratified randomization for age, gender and VO_2max_ response. CON (continuous moderate intensity) trained for another 16 weeks at moderate intensity, INC (increased intensity) trained energy-equivalent for 8 weeks at 70% HR_reserve_ and then performed high-intensity interval training (4 × 4) for another 8 weeks. Responders were identified as participants with VO_2max_ increase above the technical measurement error.

**Results:**

There was a significant difference in ${\dot{\text{V}}}$O_2max_ response between INC (3.4 ± 2.7 mL kg^−1^ min^−1^) and CON (0.4 ± 2.9 mL kg^−1^ min^−1^) after 26 weeks of training (*P* = 0.020). After 10 weeks of moderate training, in total 16 of 31 participants were classified as VO_2max_ responders (52%). After another 16 weeks continuous moderate intensity training, no further increase of responders was observed in CON. In contrast, the energy equivalent training with increasing training intensity in INC significantly (*P* = 0.031) increased the number of responders to 13 of 15 (87%). The energy equivalent higher training intensities increased the rate of responders more effectively than continued moderate training intensities (*P* = 0.012).

**Conclusion:**

High-intensity interval training increases the rate of response in VO_2max_ to endurance training even when the total energy expenditure is held constant. Maintaining moderate endurance training intensities might not be the best choice to optimize training gains.

*Trial Registration* German Clinical Trials Register, DRKS00031445, Registered 08 March 2023—Retrospectively registered, https://www.drks.de/DRKS00031445

## Key points

- Prolonging an aerobic training program after 10 weeks with constant intensity of 55% HR_reserve_ and constant energy expenditure for 16 more weeks will not increase the rate of training responders.
- Increasing training intensity to 70% HR_reserve_ at constant energy expenditure is not sufficient to further increase the rate of responders after a 10-week moderate-intensity training period.
- Training in the high intensity domain increases the rate of responders in energy-matched training interventions.

## Introduction

Endurance training is an effective way to improve cardiorespiratory fitness (CRF) and positively impact on risk factors for cardiovascular disease [1–3]. Yet, it is also known that adaptations to a standardized training greatly differ between individuals. Persons showing only minor or no adaptions to training stimuli are frequently termed nonresponders. Therefore, consideration of individual variability in training response has become increasingly important in training studies in recent years. The Heritage study was one of the first examples for large-scale intervention studies that has reported a large heterogeneity in responses of VO_2max_ after a standardized training intervention [4]. The phenomenon of little or no training response has been investigated by other researchers since then [5–7]. Ross and colleagues compared eight endurance training studies and found a range of adaptions from minus 33% to plus 118%, independent of exercise duration, intensity and trial sample size [8].

It is not clear whether adjusting the training modalities (e.g., training volume or intensity) has a positive influence on the rate of responders. The dose–response relationship of endurance training is influenced by training volume, intensity and training frequency [9–11]. Higher doses of training have proven to elicit more pronounced training adaptions [12, 13]. Furthermore, Bonafiglia and colleagues have shown that between-subject variability in training response is due to the training dose and external factors rather than interindividual differences in trainability [14]. The extent to which modifications of either intensity or volume are the leading factor is a question with large relevance to exercise training prescriptions to achieve the intended training adaptions. There is evidence that training protocols with higher intensities such as high-intensity interval training (HIIT) are more effective in eliciting increases in VO_2max_ than moderate intensity training [10, 15, 16].

Due to the high variability of adaptions to endurance training, it has been suggested that the efficacy of training interventions has to be analysed beyond mere comparison of main effects like VO_2max_ [17, 18]. In this regard, a common approach is to compare the rates of nonresponders between groups [6, 19]. In terms of interindividual variations in training response, Bonafiglia and colleagues [12] found that higher doses of training produce higher rates of response. In a direct comparison of moderate intensity training with energy-matched HIIT, Maturana and colleagues [7] found a greater effect on VO_2max_ and a lower nonresponder rate for HIIT. Montero and Lundby [19] observed that higher training dose through increased volume is an effective approach to achieve a meaningful response in VO_2max_ for participants showing no response after an initial training intervention. However, this inevitably leads to more time consumption. And since the study was lacking a control group, the question of whether extended exposure to the same training dose is sufficient to elicit a response remains unanswered. For a standardized EE, participants who performed training with higher intensities were less likely to show a nonresponse than participants training with lower intensities [6]. However, it should be noted that in this study the training frequency was 5 week^−1^ and therefore presumably higher than in most training beginners. These findings indicate that for nonresponders who are unable to perform more or longer training sessions, training with higher intensities might be a promising alternative to achieve greater and continuous training adaptions.

A methodological difficulty is the identification of nonresponders, as there is no uniform established definition of high-, low- or nonresponse. Individuals are termed responders when their individual response exceeds a certain threshold. The threshold for nonresponse has for example been as set as a VO_2max_ improvement ≤ 0 L min^−1^ [20], or less than 5% [21, 22]. Moreover, some studies use a coefficient of variation of 5.6% [5] derived from the literature. Although these thresholds are a straightforward method to categorize responders, they do not consider the true training-induced response. In this context, more attention has been given to the necessity of distinguishing the true training-induced response from within-subject variability and measurement errors of the specific setting [17]. Accordingly, some authors have suggested to use specific study designs such as repeated testing or reliability trials to analyse true training responses [23, 24]. A more individualized approach to take the day-to-day biological error and measurement error into account when interpreting and categorizing the response is calculating the technical error of measurement (TE_M_) and using it as threshold for adaptions [24, 25]. It should be noted that a dichotomous approach in which individuals are labelled as (non-)responders on the basis of a single threshold is not without criticism. A single response threshold is more likely to distinguish a “true” response from an uncertain response than a “true” nonresponse [26, 27]. Moreover, classifying subjects as nonresponders on the basis of a single parameter neglects the wide range of possible biological training adaptations [28]. Another methodological challenge in testing the independent effect of training intensity lies in the necessity to control for the overall training dose (volume × intensity). This can be achieved by estimating and standardizing the total energy expenditure (EE) between different training modalities.

In this study, we sought to compare response rates between 26 weeks of moderate intensity training (55% HR_reserve_) and training with an increase in training intensity (to 70%HR_reserve_ and to 95%HR_max_) after 10 and 18 weeks, respectively. The differences on the group level are subject in another publication and not discussed in detail here [29]. It was hypothesized that nonresponse rate can be reduced or even eliminated through an increase in intensity with energy expenditure held constant.

## Material and Methods

All participants signed written informed consent. The study procedures were in accordance with the Declaration of Helsinki and the study was approved by the Ethics Committee of the Medical Association of Saarland (identification number 219/19).

### Study Design and Randomization

Two training groups of untrained individuals performed 26 weeks of endurance training. The control group (CON) kept all training variables constant over the 26 weeks, whereas the intervention group (INC) increased training intensity after 10 and again after 18 weeks of training (for details see training below). The exercise EE was standardized on a within-subject basis by proportionally lowering the training volume in INC when the training intensity was increased. Treadmill tests to voluntary exhaustion were performed three times at baseline (T01, T02, T03), after 10 weeks (weeks 10), after 18 weeks (weeks 18) and after 26 weeks (weeks 26) of training. Using a minimization technique at week 10, subjects were allocated to either CON or INC [30, 31]. Factors for balancing were age, sex, baseline VO_2max_, VO_2max_ response at week 10 (yes/no), and the magnitude of VO_2max_ response at week 10. The objective of this procedure was to balance the amount of responders in both groups. For this reason the intraindividual variability in VO_2max_ (mL min^−1^) was used as each participant’s threshold for response [5]. The intraindividual variability (iV) represents the day-to-day variability of VO_2max_ between T02 and T03 and was calculated as follows:

$${\text{iV}} = \left| {{\text{T}}02 - {\text{T}}03} \right|/{\text{meanT02T03}}$$

### Participants

**Fig. 1:**
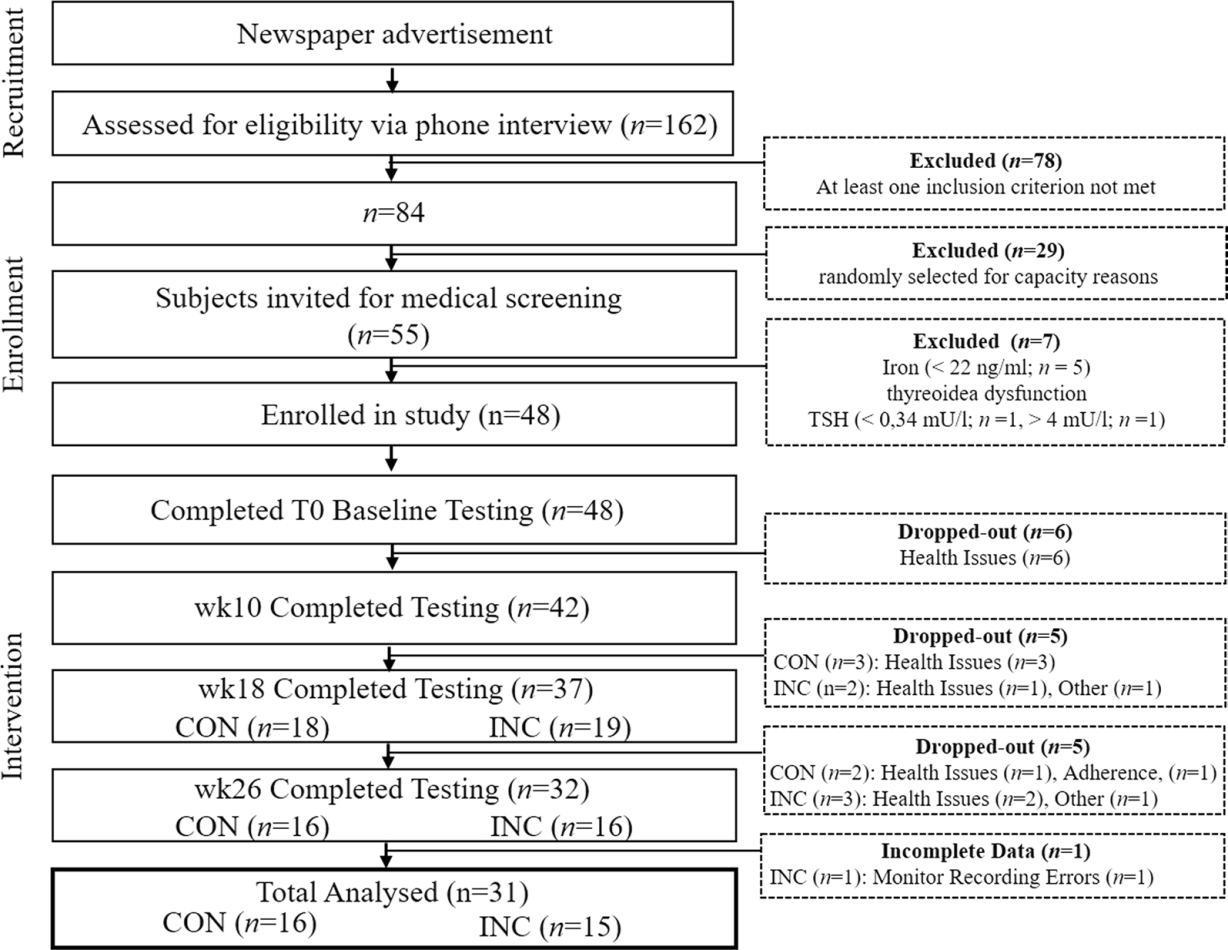
Participant flow chart. Health issues were respiratory tract infections or musculoskeletal problems (e.g. overuse injuries)

Forty-eight (48), untrained subjects were recruited for participation. Inclusion criteria were, age 30–60 years, untrained status (last 6 months: < 1 h week^−1^ endurance-type physical activity) and non-smoking (to avoid effects from cessation/reduction). Exclusion criteria were BMI > 30 kg m^−2^, resting blood pressure (RR_rest_) ≥ 160/100 mmHg, total cholesterol ≥ 300 mg dL^−1^, maximum oxygen uptake (VO_2max_) > 50 mL kg^−1^ min^−1^ for men; > 45 mL kg^−1^ min^−1^ for women, iron deficiency (Ferritin ≤ 34 ng mL^−1^), thyroid dysfunction (TSH ≤ 0.34 mU L^−1^ ≥ 4.0 ng mL^−1^), medications with potential influence on target parameters (e.g. beta-blockers) and pregnancy. Thirty-two participants (46 ± 8 years, 25.4 ± 3.3 kg m^−2^ and 34 ± 4 mL min^−1^ kg^−1^) completed the 26 weeks training programme of which one data set could not be analysed due to technical issues. A participant flow chart is given in Fig. 1. At baseline and at weeks 10, there were no between-group differences for CON and INC (*P* > 0.05). Participants’ characteristics are displayed in Table 1.

**Table 1:** Participants characteristics at baseline

	Men	Women	*P*
*n*	13	18	
Anthropometric data			
Age (years)	47 ± 9	46 ± 8	0.65
Height (cm)	176.0 ± 6.8	167.2 ± 6.6	0.01
Weight (kg)	83.9 ± 12.4	68.2 ± 11.1	0.01
BMI (kg m^−2^)	27.0 ± 2.9	24.3 ± 3.3	0.02
Body fat (%)	21.3 ± 2.9	23.6 ± 3.9	0.09
Hemodynamic characteristics at rest			
HR (bpm)	68 ± 7	69 ± 8	0.79
Systolic BP (mmHg)	124 ± 8	118 ± 9	0.06
Diastolic BP (mmHg)	83 ± 6	78 ± 8	0.04
Peak exercise performance			
VO_2max_ (mL min^−1^)	3019 ± 378	2231 ± 324	< 0.01
VO_2max_ (mL min^−1^ kg^−1^)	36.3 ± 4.1	33.1 ± 4.2	0.04
V_max_ (km h^−1^)	12.1 ± 1.1	10.4 ± 1.2	0.01
HR_max_ (b m^−1^)	186 ± 15	183 ± 11	0.47
La_max_ (mmol L^−1^)	9.3 ± 2.0	8.7 ± 2.4	0.42
RER_max_	1.2 ± 0.1	1.2 ± 0.1	0.89

Values are means ± SD; *BMI* body mass index, *HR* heart rate, *BP* blood pressure, $\dot{V}$*O*_*2max*_ maximum oxygen uptake, *V*_*max*_ maximum speed, *La*_*max*_ maximum lactate, *RER*_*max*_ maximum respiratory exchange ratio, *P* = between-group differences at baseline

### Training

Participants performed 26 weeks of walking or jogging on 3 day week^−1^. For the first 10 weeks all participants trained at moderate intensity (50 min per session at 55% HR_reserve_ [%HR_R_]). After week 10, INC increased intensity to 70% HR_R_ and to a HIIT protocol after week 18. HIIT was performed with a 10 min warm-up at 70% HR_max_ followed by 4 times 4 min at 95% HR_max_ interspersed with 3 min at 70% HR_max_ and a cool-down at the same intensity. CON trained with moderate intensity throughout the entire course of the study. The length of training sessions were adjusted to maintain a constant within-subject EE. Adherence to the prescribed training intensity was recorded with a heart rate monitor (Sigma R1 Duo + ID.Free, Sigma-Elektro GmbH, Neustadt, Germany).

### Energy Expenditure

Oxygen uptake at the individual exercise heart rates was measured during the treadmill tests and used to estimate the EE for participants exercise heart rates by use of an average caloric equivalent (4.85 kcal L^−1^ O_2_) [32]. The EE for a certain heart rate was then multiplied with the prescribed training time at that heart rate. By adjusting session length, the overall within-subject EE was kept constant in INC throughout all intervention phases. For HIIT, this implied an individual adjustment of the cool-down length.

### Testing

The first baseline test (T01) served as habituation to the maximal treadmill test and a medical examination with resting and exercise electrocardiogram (ECG). The following two baseline measurements (T02, T03) were performed to determine the intraindividual variability and the TE_M_ of ${\dot{\text{V}}}$O_2max_. The baseline data represents the mean from both tests. Before each treadmill test, anthropometric data (height, weight, BMI and body fat) as well as hemodynamic characteristics (resting heart rate and blood pressure) were taken. Body fat percentage was estimated by a 10-site skinfold method with a Harpenden caliper. Hemodynamic measures at rest were taken after a ten-minute-rest in supine position. Treadmill tests were performed on a Woodway ELG 70 (Woodway GmbH, Weil am Rhein, Germany) and gas exchange measurements were conducted continuously using a breath-by-breath system (MetaLyzer® 3B, Cortex Biophysik GmbH, Leipzig, Germany) which was calibrated according to manufacturer’s instruction.

### Treadmill test Protocol

The treadmill was set to a constant incline of 0.5%. All tests started at 4.0 km h^−1^. Every 3 min, speed was increased by 1.0 km h^−1^. The submaximal heart rate (HR_submax_) during the graded exercise test was taken at the end of last 3 min stage. When the respiratory exchange ratio (REF) exceeded 0.95 for at least 30 s this was defined as last 3 min stage. Afterwards, the tests continued using a rampwise protocol with a speed increment of 0.8 km h^−1^ per minute until voluntary exhaustion [33]. Maximal parameters were only analysed if at least two of the following criteria were fulfilled: (1) HR_max_ ≥ 220-age, (2) maximal blood lactate concentration > 8 mmol L^−1^, (3) maximal RER > 1.1 [34].

### Responder Classification

**Fig. 2:**
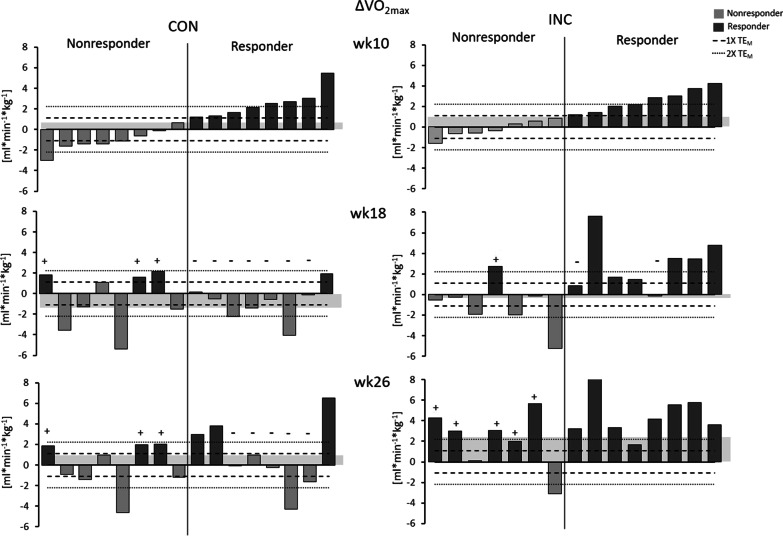
Individual changes compared to baseline in VO_2max_ [mL min^−1^ kg^−1^] after 10, 18 and 26 weeks of training for CON and INC. The through line distinguishes initial responders from nonresponders (1 × TE_M_ = single technical error, 2 × TE_M_ = multiple technical error); + eliminated nonresponse; − response turned into nonresponse. Each bar represents one subject. The order of bars is consistent in all figures. The group mean is illustrated through the bar in the background

Responders were identified after the 26-week training intervention by determining the TE_M_, which is described as a conservative measure of assessor error and day-to-day variation in conducting an exercise test [6]. This application is considered very reliable in providing an estimate of the technical error which is unaffected by a change in the mean [35]. Values ≤ 1 × TE_M_ were considered a nonresponse, > 1 × TE_M_ a response. As some authors suggest a multiple of the TE_M_ (2 × TE_M_) as a more conservative measure for response [23], these thresholds are given in Fig. 2, too. The TE_M_ was calculated by dividing the standard deviation of the difference score (*S*_diff_) by $\sqrt 2$ [35].

$${\text{TE}}_{{\text{M}}} = {{S_{{{\text{diff}}}} } \mathord{\left/ {\vphantom {{S_{{{\text{diff}}}} } {\sqrt 2 }}} \right. \kern-0pt} {\sqrt 2 }}$$

### Statistical Analyses

The sample size was estimated a priori using an effect size for comparison between groups from two out of four arms of a previous INC training intervention study by Helgerud et al. (“4 × 4” vs. “LSD”: *d*_ppc2_ = 0.547) [10]. G*Power (version 3.1.9.4) was used to calculate the sample size for an ANOVA (main and interaction effect in VO_2max_ [mL min^−1^ kg^−1^]) with *α* = 0.05, 80% power, 2 groups, 2 test time points. This led to a required sample size of *n* = 29 in total or 15 subjects per group, respectively. Taking into account an estimated drop-out rate of 28% [36], the aim was to start the study with *n* > 40 respectively *n* > 20 per group.

IBM’s Statistical Package for the Social Science (SPSS v27; IBM; USA) was used for statistical analysis. Sex differences at baseline were examined using *t*-tests for independent samples. Mann–Whitney-*U*-Tests were used to compare baseline characteristics between responders and (uncertain)nonresponders. The percentage of responders was calculated on the number of individuals who met less than 1 × TE_M_ between week 10 and week 26 for each group. Fisher's Exact Test was used to compare the rate of responders between groups at week 10, week 18 and week 26. The McNemar Test was used to compare the rates of response between week 10 and week 26 within-group. A *P* value of < 0.05 for the *α*-error was considered statistically significant.

## Results

### Compliance, Exercise Intervention and Test Criteria

Regarding frequency of training, adherence to prescribed exercise heart rate and EE, there were no statistically significant group differences (*P* > 0.05). CON completed 79.6 ± 6.95 and INC 79.8 ± 8.09 sessions. The average EE per training session estimated by indirect calorimetry was 401 ± 105 kcal with no group difference (*P* = 0.914). Average exercise HR up to week 10 was 100 ± 2% (CON) and 101 ± 2% (INC) of prescribed HR, from week 10 to week 18 it was 101 ± 4% (CON) and 99 ± 1% (INC) and from week 18 to week 26 it was 99 ± 1% (CON) and 97 ± 2% (INC). The drop-out rate was 35% (Fig. 1). HR_max_ (b min^−1^), La_max_ (mmol L^−1^) and RER_max_ indicated maximal exhaustion in both groups from baseline (CON: 186 ± 12; 9.5 ± 2.2; 1.2 ± 0.1; INC: 182 ± 13; 8.4 ± 2.1; 1.2 ± 0.1) to weeks 26 (CON: 185 ± 11; 9.5 ± 2.2; 1.3 ± 0.1; INC: 181 ± 12; 9.4 ± 2.0; 1.3 ± 0.1).

### Baseline Analysis

There were no group-differences at baseline for anthropometric data for age (*P* = 0.73), BMI (*P* = 0.95), BF (*P* = 0.41), hemodynamic characteristics at rest for HR (*P* = 0.65), BP_sys_ (*P* = 0.44), BP_dias_ (*P* = 0.29) and peak exercise data for relative VO_2max_ (*P* = 0.57), absolute VO_2max_ (*P* = 0.71), V_max_ (*P* = 0.50), HR_max_ (*P* = 0.44), La_max_ (*P* = 0.17) and RER_max_ (*P* = 0.52). Baseline characteristics are shown in Table 1. There were no differences between responders and nonresponders at baseline for age (*P* = 0.06), BMI (*P* = 0.62), HR_rest_ (*P* = 0.65), BP_sys_ (*P* = 0.32), BP_dias_ (*P* = 0.38), relative VO_2max_ (*P* = 0.98), absolute VO_2max_ (*P* = 0.86) and V_max_ (*P* = 0.86).

### Mean Responses

Baseline data as well as changes over time for all parameters are presented in Table 2.

**Table 2:** Baseline values and changes after 10, 18 and 26 weeks of training for group and response

	Baseline	*Δ* weeks 0–10	*n* (%)	*Δ* weeks 10–18	*n* (%)	*Δ* weeks 18–26	*n* (%)
VO_2max_ [mL min^−1^ kg^−1^]							
CON							
r	34.9 ± 3.5	2.5 ± 1.4	[8 (50)]	1.5 ± 3.5	[4 (25)]	2.0 ± 2.2	[6 (37)]
nr	34.9 ± 3.9	− 1.1 ± 1.1	[8 (50)]	− 2.4 ± 2.3	[12 (75)]	0.7 ± 1.3	[10 (63)]
INC							
r	33.0 ± 4.7	2.6 ± 1.1	[8 (53)]	1.3 ± 2.5	[7 (47)]	2.4 ± 2.0	[13 (87)]
nr	35.0 ± 5.9	− 0.2 ± 0.8	[7 (47)]	− 1.5 ± 2.3	[8 (53)]	2.1 ± 0.1	[2 (13)]
VO_2max_ [mL min^−1^]							
CON							
r	2595 ± 601	167 ± 15	[6 (37)]	171.0 ± 39.6	[2 (12)]	139.3 ± 144.7	[4 (25)]
nr	2596 ± 542	− 54 ± 93	[10 (63)]	− 151.4 ± 175.1	[14 (88)]	47.8 ± 120.5	[12 (75)]
INC							
r	2645 ± 531	145 ± 64	[9 (60)]	78.7 ± 124.4	[6 (40)]	123.0 ± 142.2	[11 (73)]
nr	2343 ± 473	− 3 ± 60	[6 (40)]	− 162.1 ± 125.9	[9 (60)]	116.8 ± 129.9	[4 (27)]
V_max_ [km h^−1^]							
CON							
r	11.2 ± 1.4	0.8 ± 0.3	[15 (94)]	0.2 ± 0.3	[16 (100)]	0.0 ± 0.3	[16 (100)]
nr	13.2 ± 0.0	0.4 ± 0.0	[1 (6)]	/ ± /	[0]	/ ± /	[0]
INC							
r	10.8 ± 1.5	0.8 ± 0.3	[14 (93)]	0.5 ± 0.4	[14 (93)]	0.5 ± 0.4	[14 (93)]
nr	12.2 ± 0.0	− 0.4 ± 0.0	[1 (7)]	0.2 ± 0.0	[1 (7)]	− 0.6 ± 0.0	[1 (7)]
HR_rest_ [b m^−1^]							
CON							
r	74.4 ± 9.2	− 9.0 ± 6.8	[7 (44)]	− 0.7 ± 5.8	[6 (38)]	− 1.6 ± 5.2	[4 (25)]
nr	65.3 ± 6.6	− 1.0 ± 1.6	[9 (56)]	3.5 ± 3.6	[10 (62)]	1.2 ± 3.0	[12 (75)]
INC							
r	70.6 ± 4.2	− 8.2 ± 2.3	[5 (33)]	− 7.2 ± 5.7	[6 (40)]	− 3.0 ± 3.9	[13 (87)]
nr	66.7 ± 6.3	2.3 ± 4.1	[10 (67)]	− 0.3 ± 5.7	[9 (60)]	− 1.0 ± 2.8	[2(13)]
HR_submax_ [b m^−1^]							
CON							
r	137.3 ± 7.7	− 7.4 ± 3.1	[8 (50)]	− 1.5 ± 5.7	[8 (50)]	− 0.5 ± 4.1	[10 (63)]
nr	134.6 ± 16.0	− 2.7 ± 2.3	[8 (50)]	2.1 ± 8.6	[8 (50)]	1.4 ± 4.9	[6 (37)]
INC							
r	132.2 ± 15.6	− 10.8 ± 3.3	[7 (47)]	− 3.5 ± 5.5	[10 (67)]	− 2.0 ± 7.6	[10 (67)]
nr	132.7 ± 8.8	4.2 ± 3.8	[8 (53)]	− 4.4 ± 5.7	[5 (33)]	0.9 ± 7.5	[5 (33)]

*Δ* = changes after 10, 18 and 26 weeks of training for CON and INC in VO_2max_ [mL min^−1^ kg^−1^]; VO_2max_ [mL min^−1^], V_max_ [km h^−1^]; HR_rest_ [b min^−1^]; HR_submax_ [b min^−1^]; values are presented as means ± SD; r = responder, nr = nonresponder; *n* (%) = number (rate) of responders/nonresponders

After 26 weeks of training INC improved ${\dot{\text{V}}}$O_2max_ significantly by 3.4 ± 2.7 mL kg^−1^ min^−1^ (*P* = 0.002) as well as from week 18 to week 26 (*P* = 0.002). Changes in CON were not significant (0.4 ± 2.9 mL kg^−1^ min^−1^). For relative VO_2max_, improvements were significantly higher for INC than for CON (*P* = 0.02). The mean values for relative VO_2max_ at baseline and after weeks 10, weeks 18 and weeks 26 for CON were 34.9 ± 3.6, 35.6 ± 4.5, 34.1 ± 3.9, 35.3 ± 4.0 mL kg^−1^ min^−1^ and for INC were 34.0 ± 5.2, 35.2 ± 5.5, 35.0 ± 5.1, 37.4 ± 4.9 mL kg^−1^ min^−1^, as described elsewhere in more detail [29].

### Rate of Responders

Changes in VO_2max_ after 10, 18 and 26 weeks of training for CON and INC are shown in Table 2. INC increased the rate of responders more effectively than CON (*P* = 0.012).

After 10 weeks of training at an intensity of 55%HR_R_, there were no between-groups differences in the rate of responders for relative VO_2max_ (CON: 50%; INC: 53%; *P* = 0.569), HR_rest_ (CON: 56%; INC: 33%; *P* = 0.179) and HR_submax_ (CON: 50%; INC: 47%; *P* = 0.569).

After 18 weeks of training there were also no between-groups differences in the rate of responders for relative VO_2max_ (CON: 25%; INC: 47%;* P* = 0.189), HR_rest_ (CON: 62%; INC: 40%; *P* = 0.186) and HR_submax_ (CON: 50%; INC: 67%; *P* = 0.283).

After 26 weeks of training the differences in the rate of responders were greater for INC than for CON for relative VO_2max_ (CON: 37%; INC: 87%;* P* = 0.009) and HR_rest_ (CON: 25%; INC: 87%; *P* < 0.001) but not for HR_submax_ (CON: 63%; INC: 67%; *P* = 0.553).

Within group analysis showed that the rate of response was increased between week 10 and week 26 in INC for relative VO_2max_ from 53 to 87% (*P* = 0.063) and HR_rest_ from 33 to 87% (*P* = 0.008). From week 18 to week 26 INC increased the rate of VO_2max_ responders from 47 to 87% (*P* = 0.031).

In CON there was a non-significant decrease in the rate of response for relative VO_2max_ from 50 to 37% (*P* = 0.754) and HR_rest_ from 44 to 25% (*P* = 0.25) from week 10 to week 26.

## Discussion

This study investigated the role of an increase in training intensity while maintaining EE for increasing the responder rate to endurance training in healthy untrained subjects. With an average EE per training session of 400 kcal, positive effects can be achieved by both moderate and intensive training. As expected, a high interindividual variation of the response was observed. Responders for VO_2max_ were observed in both groups and by stratified randomization equally distributed (CON = 50%; INC = 53%). The main findings of this research are (1) through an increase in training intensity response rate for VO_2max_ was increased from 53 to 87% despite total EE remaining the same, (2) given a constant energy consumption, a high intensity interval training effectively increases the rate of responders as indicated by an additional increase in response rate between week 18 and week 26 (it should be noted that this increase occurred after 18 weeks of training had already been completed) and (3) the training stimulus at an intensity of 70%HR_R_ is not sufficient to increase response after 10 weeks of training at 55%HR_R_.

We were able to show that for standardized isocaloric training interventions the choice of intensity affects the magnitude of ΔVO_2max_ as well as the rate of response. When training intensity was increased from 55%HR_R_ to 70%HR_R_ between week 10 and 18 weeks no increase in response rate was observed after 8 weeks. Only when INC performed HIIT between week 18 and week 26, the response rate was significantly increased (+ 75%) in most participants. This suggests that for isocaloric interventions the intensity must be in the high intensity domain to produce more responders. Previous studies have already shown that HIIT is more effective than moderate training for improvements of CRF when EE is controlled for [37, 38]. A novel finding of our study is that there does not seem to be a linear relationship between intensity and responder rates as training with 70%HR_R_ did not increase response rate effectively. It is likely that certain physiological pathways leading to higher VO_2max_ are activated particularly through a training stimulus above a certain threshold. There is evidence that HIIT yields greater improvements in mitochondrial content than work-matched moderate training [39]. Torma and colleagues related the higher effectiveness of HIIT to a larger activation of fast-twitch fibres and an advanced mitochondrial biogenesis [40]. Despite these works it is still unclear what the exact pathways are that lead to higher VO_2max_. It seems unlikely that even for a standardized training programme responders improve their ΔVO_2max_ through a uniform physiological adaption process [41]. Although our data show that HIIT is more likely to trigger a response in markers of CRF, there were still 2 out of 8 initial nonresponders in INC after 26 weeks. This agrees with findings of Timmons and colleagues [42] that 20% of individuals show no improvement in aerobic capacity even after high intensity training. Considering Montero’s and Lundby’s [19] study which shows that nonresponse can be eliminated through training doses of up to 300 min per week, we conclude that for some individuals an adjustment to HIIT for a given training dose might not be sufficient and higher overall training doses, i.e. higher EE, are necessary in these cases to elicit a training response increase response.

The overall effects on VO_2max_ as well as the rate of responders after the first 10 weeks of CON were comparable with other studies investigating the effects of moderate intensity training [10, 43]. Over the next 16 weeks, 3 additional participants, from an initial 8, became responders. From the initial 8 responders at baseline 7 were categorized as nonresponders (respectively uncertain responders) at weeks 18 and 5 at week 26. Our data therefore suggest that continued training at the same intensity does not automatically have a positive effect on the rate of responders. Additionally, in accordance with international guidelines [44], we found that moderate intensity training might not always produce a lasting training response when the training dose is not intensified over time. One rationale for this finding is that the first 10 weeks of training triggered primarily adaptions of the cardiovascular system that led to improved mechanisms of oxygen transport and consumption and therefore an increase in VO_2max_. These adaptions plateaued and, in some cases, possibly even regressed when the training dose remained the same and submaximal adaptions (e.g. metabolic system, running economy) presumably became more pronounced over time. In this context, it must be emphasized that the exercise heart rate was not significantly adjusted in CON at any point over 26 weeks and effectively increased over time. This may have caused a lower metabolic strain over time for a given heart rate because of higher metabolic efficiency, which could cause a decreasing training stimulus over time for these individuals. This assumption is supported by findings by Weatherwax and colleagues who compared response rates for a training prescription based on %HR_R_ with an individualized training based on ventilatory thresholds [11]. The response rates were 60% for the standardized and 100% for the individualized training. In line with these findings and others [45, 46] the question remains whether response rates indicate differences in trainability or are a consequence of variation in the between-subject metabolic strain for a fixed heart rate percentage. Although the literature shows that individualized training prescription can produce more homogenous training responses [47], it should not be overlooked that in many practical settings the necessary metabolic testing for that type of prescription is not feasible. For the time being, the majority of individuals that take up an unsupervised exercise regimen will rely on the international guidelines and use heart rate as the only physiological measure to monitor training intensity. In this context, our study has shown that the implementation of high intensity efforts increases the likelihood of improved fitness.

Although the magnitude of adaption for V_max_ between the two groups were in favour of INC, there were no difference in the response rates for this parameter. This highlights that moderate as well as intense training protocols yield improvements in functional capacity. Vollaard and colleagues have already shown that for untrained individuals improvements in maximal running speed are not related to VO_2max_ [48]. Our study has confirmed these findings, as there was no uniform pattern of response for V_max_ and VO_2max_. Although the prognostic value of VO_2max_ for overall health and all-cause mortality is widely accepted, it is still unclear to which degree VO_2max_ or the higher levels of physical activity through which it is achieved are the causal factor for improved health [49]. We therefore suggest future research to focus more on the connection between nonresponse and health benefits, acknowledging the methodical challenges in such research designs. In this context it should be noted that an adaptation below a certain threshold is not equal to a nonresponse as there is a range of uncertainty between “true” response and nonresponse.

### Limitations

Our data point out that a single post-test might not be sufficient to categorize individuals as responders with certainty. We have shown that for a standardized training intervention there is a considerable within-subject fluctuation of response over time. This is in line with other recommendations for repeated testing during an exercise intervention [23, 24]. Nevertheless, the strength of this study is that the within-subject variability of VO_2max_ was analysed by two baseline measurements. In our study, the TE_M_ was 4.2 ± 2.7% and was comparable in both groups. Thus, it was similar to the often used coefficient of variation of 5.6% reported in literature [5]. In other studies, most subjects showed a response in at least one parameter, except for VO_2max_ [5]. This highlights that the efficacy of training interventions should be assessed through several measures.

Since the level of intraindividual variability may have an influence on the response, it would be interesting to see how intraindividual variability changes in the training process. For this purpose, Ross and colleagues [8] recommend including a control group to measure the interindividual response variability and to perform additional measurements at the pre- and post-tests. Participants were instructed to maintain eating habits as well as daily physical activity. As we did not record the daily physical activity, we cannot rule out tiny influences from this source and suggest future research to include the influence of lifestyle habits on the training response.

## Conclusion

The current study demonstrates that training in the high intensity domain increases the rate of responders for VO_2max_ for most individuals even when the total energy expenditure remains the same. Thus, training at intensities around VO_2max_ such as in HIIT is well suited to effectively increase the rate of responders. If the specifications of a standardized moderate endurance training remain constant after the period of 10 weeks of training, further training adaptations are unlikely. Therefore, training in the high intensity domain is an effective way to achieve and maintain a training response without the need of a higher total training volume. This is of particular relevance for settings, where individualized trainings prescriptions are not feasible.

## Data Availability

The datasets used and/or analysed during the current study are available from the corresponding author on reasonable request.

## Abbreviations

BMI: Body mass index
BP: Blood pressure
CON: Control group (moderate intensity)
CRF: Cardiorespiratory fitness
EE: Total energy expenditure
HIIT: High-intensity interval training
HR_rest_: Resting heart rate
HR_reserve_: Heart rate reserve
HR_submax_: Submaximal heart rate
INC: Intervention group (increasing intensity)
iV: Intraindividual variability
La_max_: Maximum lactate
RER: Respiratory exchange ratio
T01: Habituation to the maximal treadmill test and a medical examination
T02: Baseline measurement 1
T03: Baseline measurement 2
TE_M_: Technical error of measurement
V_max_: Maximum speed
VO_2max_: Maximum oxygen uptake
wk10: 10 Weeks of training
wk18: 18 Weeks of training
wk26: 26 Weeks of training
